# Coexistence of Scattering Enhancement and Suppression by Plasmonic Cavity Modes in Loaded Dimer Gap-Antennas

**DOI:** 10.1038/srep17234

**Published:** 2015-11-27

**Authors:** Qiang Zhang, Jun-Jun Xiao, Meili Li, Dezhuan Han, Lei Gao

**Affiliations:** 1 College of Electronic and Information Engineering, Shenzhen Graduate School, Harbin Institute of Technology, Xili, Shenzhen 518055, China; 2 Department of Applied Physics, Chongqing University, Chongqing 400044, China; 3 College of Physics, Optoelectronics and Energy of Soochow University, & Collaborative Innovation Center of Suzhou Nano Science and Technology, Soochow University, Suzhou 215006, China; 4 Jiangsu Key Laboratory of Thin Films, Soochow University, Suzhou 215006, China

## Abstract

Plasmonic nanoantenna is of promising applications in optical sensing and detection, enhancement of optical nonlinear effect, surface optical spectroscopy, photoemission, etc. Here we show that in a carefully-designed dimer gap-antenna made by two metallic nanorods, the longitudinal plasmon antenna mode (AM) of bonding dipoles can compete with the transverse plasmonic cavity modes (CMs), yielding dramatically enhanced or suppressed scattering efficiency, depending on the CMs symmetry characteristics. More specifically, it is demonstrated that an appropriately loaded gap layer enables substantial excitation of toroidal moment and its strong interaction with the AM dipole moment, resulting in Fano- or electromagnetically induced transparency (EIT)-like profile in the scattering spectrum. However, for CMs with nonzero azimuthal number, the spectrum features a cumulative signature of the respective AM and CM resonances. We supply both detailed near-field and far-field analysis, showing that the modal overlap and phase relationship between the fundamental moments of different order play a crucial role. Finally, we show that the resonance bands of the AM and CMs can be tuned by adjusting the geometry parameters and the permittivity of the load. Our results may be useful in plasmonic cloaking, spin-polarized directional light emission, ultra-sensitive optical sensing, and plasmon-mediated photoluminescence.

Plasmonic nanoparticles and their assemblies are well-known optical nanoantennas, and have been intensively studied in nanophotonics due to the fascinating optical properties originated from localized surface plasmon resonance (LSPR)^1^,^2^. Among the various kinds of metallic nanostructures, plasmonic dimer antennas (PDAs) which are often constructed by a pair of strongly interacting metallic nanoparticles, e.g., a dimer consisting of two arms and a gap between them, have been frequently studied^3^,^4^,^5^. Despite the numerous similarities with traditional RF antenna in terms of collecting and emitting electromagnetic wave^6^,^7^, the LSPRs in PDA offer tremendous extraordinary and anomalous optical responses such as strong light confinement and scattering^8^,^9^,^10^,^11^. Moreover, the resonance features of a PDA are intimately related to the size, shape, the dielectric functions of the compositions, and the ambient enviroment^12^,^13^. The virtues of LSPR grant PDA promising applications in single molecule detecting^14^, enhanced light-matter interaction^15^, optical nano-circuit^16^,^17^, enhancement of optical nonlinear effects^18^,^19^, and plasmon-assisted particle trapping and micromanipulations^20^,^21^ etc. In addition, since PDAs represent one of the simplest coupling systems, they are quite suitable for studying plasmon hybridizations and coherent plasmonic phenomena such as Fano resonance and electromagnetically induced transparency (EIT)^22^,^23^,^24^,^25^.

To this end, a great deal of attention has been focused on the antenna mode (AM) of a PDA which is basically an electric dipolar plasmon resonance sustained by the solid metallic parts. The nanogap of a PDA provides the feeding port to excite the AM or to tune the equivalent circuit property^10^,^13^,^16^,^26^. However, in such plasmonic nanostructures, not only the solid metallic parts but also their inverse counterparts (e.g., the dielectric gap layer embedded between the metals) play significant roles. The LSPRs associated with the solid metallic parts usually give rise to prominent electric resonances while the inverse parts favor magnetic resonances, according to the Babinet principle^27^,^28^,^29^. It is more fundamental to realize that PDAs are actually composite structures with the arms being the solid part and the dielectric gap being the inverse part, simultaneously^30^,^31^,^32^,^33^,^34^,^35^. Different to the AM, cavity modes (CMs) are strongly confined in the dielectric layer with small modal volume and high quality factor^31^,^32^. In view of the multipole expansions, the lower-order CMs give rise to the fundamental magnetic multipoles^34^,^35^. More interesting, one of the CMs has been shown to generate remarkable toroidal dipole response. The toroidal dipole response could yield interesting consequences such as the formation of anapole^36^, toroidal induced transparency^37^,^38^,^39^, and enhanced optical scattering force^40^. In this context, one would expect that a PDA may support both AM and CMs in the same band and it shall be of great interest to explore the couplings between them. We note that they were respectively analyzed in detail in a very recent work^41^. However, studies on the AM-CM coupling effects are still missing.

In this study, we examine the AM-CM coupling effects by carefully adjusting the geometry and the gap layer material in a PDA consisting of two identical silver nanorods. By deliberately making the AM and CMs spectrally overlapped, we are able to simultaneously or selectively obtain destructive and cumulative responses from them, depending on the angular symmetry of the CMs. Both far-field and near-field characteristics of the designed PDAs are examined by full-wave simulations based on the finite integral technique (FIT)^42^. The results show that the magnetic dipolar CM fully decouples from the electric bonding dipolar AM and they collectively generate accumulative scattering enhancement. On the contrary, the toroidal dipolar CM violently competes with the AM in the near field, yielding Fano resonance or EIT-like features in the spectrum.

## Results

### Coupling between the antenna dipolar mode and the cavity modes

**Figure 1:**
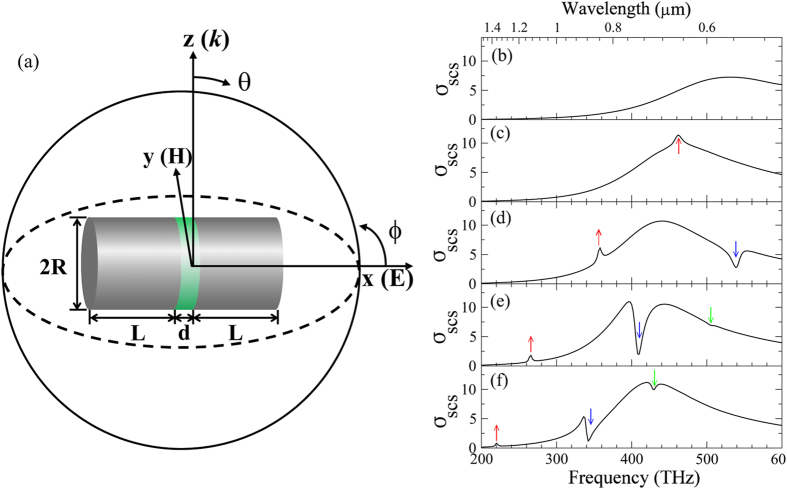
Schematic of the plasmonic dimer antenna and typical scattering spectra. (**a**) Schematic figure of the antenna and the excitation configuration. The scattering efficiency spectrum *σ*_*SCS*_ of a PDA with *L* = 90 nm, *R* = 50 nm, and *d* = 20 nm for (**b**) *ε*_*load*_ = 1, (**c**) *ε*_*load*_ = 3, (**d**) *ε*_*load*_ = 6, (**e**) *ε*_*load*_ = 12, and (**f**) *ε*_*load*_ = 18.

Figure 1a schematically shows the PDA structure which is a typical homodimer with two identical silver nanorods loaded with a dielectric layer of thickness *d* inside the gap. The two cylindrical nanorods are both of radius *R* and length *L*. The dielectric constant of the gap material is set to be *ε*_*load*_ and that of the silver is taken from Johnson and Christy^43^. The whole structure is assumed to be freestanding in air and illuminated by a normally incident plane wave (**k** along 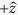 direction) that is linearly polarized along the *x* axis. Firstly, we set *R* = 50 nm, *L* = 90 nm, *d* = 20 nm and check how the scattering spectrum evolves with various *ε*_*load*_. The calculated optical scattering efficiency *σ*_*scs*_ for *ε*_*load*_ = 1, 3, 6, 12 and 18 are shown in Fig. 1b–f. It is seen that for *ε*_*load*_ = 1 the spectrum has a broad peak centered at *f* ≈ 530 THz (see Fig. 1b). This peak corresponds to the electric dipolar AM resonance which is also known as the bonding hybridization resonance from the individual dipolar modes on the two nanorods^22^. As the gap dielectric constant increases to *ε*_*load*_ = 3 (Fig. 1c), a narrow Lorentz-shape peak (marked by the red upward arrow) is superimposed on the AM peak which red shifts to *f* = 462 THz. With loaded dielectric material of *ε*_*load*_ = 6, it is seen in Fig. 1d that the narrow peak (*f* = 358 THz) resides at the left shoulder of the broad AM resonance. More interestingly, another resonance feature shows up near *f* = 540 THz, apparently with a quite different flavor. This resonance reduces rather than adds up to the AM resonance profile. It also leads to an asymmetric line shape with a Fano-like dip, as marked by the downward blue arrow in Fig. 1d. Following this trend, when *ε*_*load*_ is continuously increased to 12, which is the situation shown in Fig. 1e, both the upward-arrow-marked peak and downward-arrow-marked dip red shift. However, their relative magnitudes with respect to the AM background (envelope) exhibit different changing characteristics. The strength of the Lorentzian peak nearly does not change while that of the Fano dip visually increases as it approaches the resonance center frequency of the AM. It is seen that the original AM peak becomes a deep EIT-like dip and splits into two separate peaks at *f* = 398 THz and 444 THz, respectively. This indicates a strong interaction of the AM with another resonance mode (we will later show that it is a CM with toroidal response). Notice that in Fig. 1e, there is another tiny Fano dip at *f* ≈ 505 THz (marked by the downward green arrow) which must come from a higher-order mode. Finally, Fig. 1f shows that for *ε*_*load*_ = 18, this high-energy dip looks apparent as it now spectrally approaches the AM resonance center frequency.

**Figure 2:**
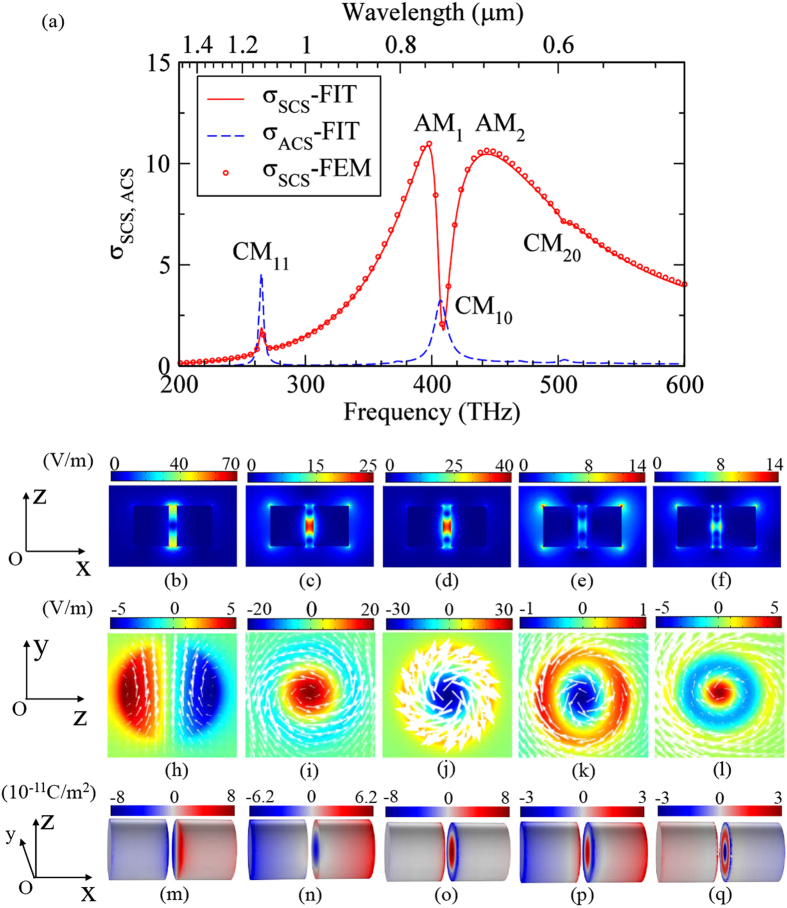
Optical spectra and the near-field details for different frequency. (**a**) The *σ*_*SCS*_ (red line) and *σ*_*ACS*_ (blue dashed line) spectra of the PDA with *L* = 90 nm, *R* = 50 nm, *d* = 20 nm and *ε*_*load*_ = 12. The red dots are results calculated by the FEM. (**b**–**f**) The electric field amplitude **|E|** at *xoz* plane for CM_11_, AM_1_, CM_10_, AM_2_ and CM_20_ in order. (**h**–**l**) The out-of-plane electric fields *E*_*x*_ (colors) and the in-plane magnetic field (arrows) at the *yoz* plane across the dielectric layer. (**m**–**q**) The corresponding surface charge distribution.

It is reasonably to infer from the above observations that the sub-features in the spectra, e.g., the peaks and the Fano-like dips, may come from another family of resonances that are distinct from the prominent AM. To clarify that, we focus on the PDA structure studied in Fig. 1e. Figure 2a shows the absorption efficiency *σ*_*ACS*_ together with the scattering efficiency *σ*_*SCS*_ of the whole structure. The FIT results were further corroborated by a solver (COMSOL Multiphysics) of finite element method (FEM)^44^. It is seen that the FEM results (symbol) agree very well with the FIT ones (red line). Different to *σ*_SCS_ whose envelop is a broad resonance spectrum with small quality factor, the *σ*_ACS_ shows three narrow peaks at *f* = 265 THz, 408 THz, and 505 THz with high quality factors. This is because that the *σ*_SCS_ of the PDA is dominated by the electric dipolar AM with large radiation loss while the *σ*_ACS_ substantially reflects the CMs with Ohmic loss caused by the induced localized currents in the metallic arms close to the gap.

To identify these resonances, we show in Fig. 2b–f the electric field amplitude 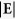 over the *xoz* plane for *f* = 265 THz, 398 THz, 409 THz, 444 THz and 505 THz, respectively. It is seen that at *f* = 265 THz, the electric field is basically confined in the gap region with a zero-field node in the center (see Fig. 2b). This strongly suggests that it originates from a plasmonic cavity mode. Figure 2h shows the snapshot of the out-of-plane electric field *E*_*x*_ (see the color contour) in the dielectric layer across the origin. We label this resonance as ‘CM_11_’ where the subscript represents the radial and azimuthal node numbers in the *E*_*x*_ pattern. Furthermore, the *yoz* in-plane magnetic field vectors are shown by arrows which exhibit a large magnetic moment along the *y* direction. This magnetic moment is formed by the anti-directional going conduction currents on the opposite gap surfaces, given by the so called magnetic resonance^30^.

Figure 2c shows that for the dominated scattering peak at *f* = 398 THz at the lower-frequency side of the Fano dip, there are hot spots both inside the gap region and near the antenna terminals. Strong electric field around the antenna terminals is exactly a signature of the AM. In fact, here it is identified as an electric dipolar resonance that strongly radiates, despite of the slight asymmetric characteristic along the *z* axis due to retardation. At the same time, Fig. 2c shows that the electric field is also much stronger inside the gap, with the zero-field node appearing near the lateral boundary. Figure 2i indicates that at this frequency the PDA also sustains the CM_10_ resonance in the gap region. These imply hybridization between the dipolar AM and the cavity mode CM_10_. For convenience, here we label the peak by ‘AM_1_’ to distinguish it from the other hybridized mode ‘AM_2_’ that contributes to the second overwhelming scattering peak at *f* = 444 THz. Figure 2d shows the electric field pattern for the scattering dip at *f* = 409 THz that falls in between AM_1_ and AM_2_. Obviously, the field around the antenna terminals supposedly produced by the AM nearly vanishes (see Fig. 2d) compared to that of AM_1_ (Fig. 2c) and AM_2_ (Fig. 2e). We note that this vanishing AM field highlights one of the important features of Fano resonance: the “bright” mode excitation is substantially suppressed at the Fano dip frequency^24^. In a similar manner, we label this resonance as ‘CM_10_’ according to the *E*_*x*_ field pattern shown in Fig. 2j wherein the in- plane magnetic field is in a circular form confined in the gap region. Such magnetic field confinement gives rise to a toroidal dipole response, as observed in circular patch metal-dielectric-metal (MDM) antennas^34^,^35^. For frequency *f* = 444 THz beyond the Fano dip, the scattering peak from ‘AM_2_’ emerges. Figure 2e shows that the hot spots around the antenna terminals recover to some extent (the frequency is away from the AM resonance center frequency) but those inside the gap diminish. The near field maps for the high-energy minor dip at *f* ≈ 505 THz are shown in Fig. 2f,l. Both of them help to confirm that the gap mode at this frequency is ‘CM_20_’.

To see more properties of these modes, we additionally examined the resonance-induced surface charges. Figure 2m–q plot the surface charge distribution for the labeled peak and dip frequencies in Fig. 2a. Overall, the induced charges at the two arms of the PDA are basically of opposite sign because that the whole frequency band is covered by the bonding dipolar AM resonance. However, the fine structures of the charge distributions on the opposing interfaces across the gap are determined by the respective CM resonance. For example, Fig. 2m shows that for CM_11_, the positive and negative charges on the two inner interfaces across the gap separate along the *y* axis, with a neutral node around the origin. Meanwhile, Fig. 2n–p show that for AM_1_, CM_10_, and AM_2_, the induced opposite charges around the gap distribute alternatively in the radial direction without azimuthal zero node. We note that this “positive-negative” ring-like charge distribution is very similar to the reported fine structure in nanosphere heterodimers at the Fano dip frequency^23^. Such distribution to some extent is ascribed to the gap cavity between the metallic arms. Indeed, the suppression of the AM at the Fano dip is more clearly seen in Fig. 2o where the metallic arms are mostly neutralized in the terminals and the surface charges accumulate near the gap interfaces. Figure 2q shows that for the CM_20_ resonance, the charges near the gap have connected ring-like pattern.

As a matter of fact, there are several other CMs in this frequency band with undistinguishable peak in the *σ*_*SCS*_ spectrum due to the weak excitation (see Supporting Information, Fig. S1). To make our discussions more concise and simple, hereafter, we ignore them and the CM_20_ mode, and specifically focus on the CM_11_ and CM_10_ modes. Figure 2h,j demonstrate that the CM_11_ and the CM_10_ have quite different symmetry pattern. It is thus expected that their modal overlapping with the AM must be distinct, yielding weak and/or strong mutual interactions, respectively. Note that a pure dipolar AM has relatively homogenous electric field in the gap region, with approximately zero azimuthal quantum number (*m* = 0). As a consequence, the field overlap integral between CM_nm_ and AM is determined by 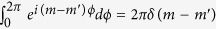, here *δ* denotes the Dirac delta function. It is immediately clear that CMs with *m* = 0 have nonzero modal overlapping with the AM, otherwise they are orthogonal to the AM. This argument holds as long as both the AM and CMs have negligible in-plane electric field.

The AM and CM can be excited by the different component of the incoming wave (**E** or **H**), therefore different *σ*_*SCS*_ spectra can be obtained in other configurations of incident plane wave (see Supporting Information, Fig. S2a). The same features can be also seen in the radiation decay rate of an electric dipole source (see Supporting Information, Fig. S2b).

**Figure 3:**
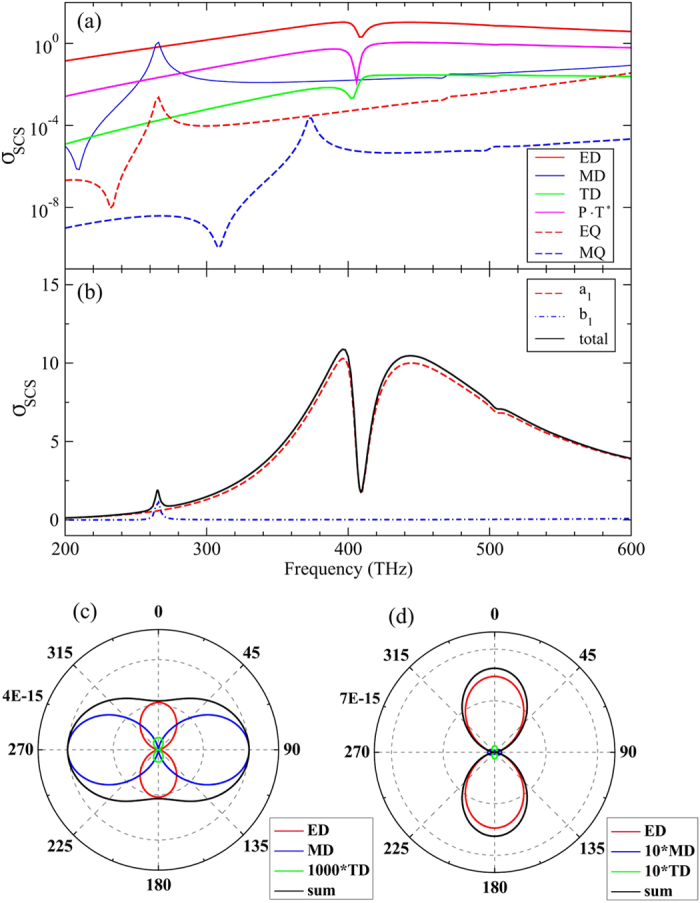
Decomposed scattering from fundamental moments and their radiation pattern. (**a**) The scattering efficiency *σ*_*SCS*_ of the irreducible multipoles in the Cartesian coordinates. (**b**) The scattering efficiency *σ*_*SCS*_ of the spherical dipole related to *a*_1_ and *b*_1_ and the total *σ*_*SCS*_. (**c**) The radiation pattern of ED, MD, TD and their summation in the *θ* = 90° plane for the CM_11_ resonance at *f* = 265 THz. Note that the far field intensity from the TD is artificially amplified 1000 times to increase the visibility. (**d**) Same as (**c**) but for the CM_10_-mode induced Fano dip at *f* = 409 THz. The MD and TD term are multiplied by 10.

It is now unambiguous that the CM_11_ and the CM_10_ have quite different interaction with the AM. The CM_11_ mode shows no considerable effect on the AM, but simply presents a cumulative peak in the scattering spectrum (Fig. 1). On the other hand, the CM_10_ mode interacts strongly with the AM, leading to a Fano (or EIT-like) dip (see Fig. 2a). More importantly, the interaction strongly suppresses the AM in the near fields due to destructive competition on the surface charge distribution, as demonstrated in Fig. 2d,o. To gain deeper understanding of the underlying physics, we have performed multipole decompositions of the total scattering fields for the PDA structure in Fig. 2. Figure 3a shows the *σ*_SCS_ from the irreducible Cartesian multipoles that include the electric dipole (ED), the magnetic dipole (MD), the toroidal dipole (TD), the ED-TD cross term notated as ‘P · T^*^’(see the Supporting Information), and the electric quadrupole (EQ) and magnetic quadrupole (MQ)^36^,^37^,^38^,^39^,^40^. It is seen in Fig. 3a (note that the *y*-axis is in logarithmic scale) that the *σ*_SCS_ of the ED (red line) dominates and leads to the broad AM resonance envelope of the total *σ*_SCS_ shown in Fig. 2a. It is therefore reasonable to consider that the AM mode gives rise to the ED which is relatively bright, namely efficiently excitable and radiating. More importantly, at the CM_10_ resonance *f* = 409 THz, the scattering efficiency from ED shows a dip. This is a direct proof that the AM is suppressed by the Fano resonance induced by the coupling between the AM and CM_10_ mode that is absent in a typical and commonly encountered PDA. The *σ*_SCS_ of the MD (see the blue line in Fig. 3a) is much smaller than that of the ED with an exception near *f* = 265 THz that is exactly the resonance frequency of the CM_11,_ indicating that the CM_11_ gives rise to a MD response, consistent with the preceding discussions. Different to the MD, the TD scattering efficiency (green line in Fig. 3a) in the whole frequency band is at least two orders of magnitude smaller than that from the ED, even near the resonance frequency of CM_10_ (*f* = 409 THz). We note that in most cases the scattering ability of a TD is indeed much weaker than an ED^39^,^40^. Figure 3a further shows that the scattering from EQ and MQ is relatively negligible. However, we can infer that there is in fact a CM resonance giving rise to MQ response at *f* ≈ 374 THz (see blue dashed line in Fig. 3a and Fig. S1 in the Supporting Information).

Figure 3b shows the decomposed scattering spectra from multipole moments in the spherical coordinate (see Supporting Information) and the total *σ*_*SCS*_ (solid black line). The scattering efficiency from spherical scattering coefficients *a*_1_ and *b*_1_ are the main contributions: their sum basically recovers the total *σ*_*SCS*_. We would like to emphasize that the *σ*_SCS_ related to *a*_1_ contains both the ED and TD parts in Fig. 3a 36,40,45. We further note that the scattering dip in the *σ*_*SCS*_ spectrum is essentially different to the recently reported toroidal induced transparency which is basically ascribed to the scattering cancellation between the ED and TD^36^,^37^,^38^. The scattering cancellation demands comparable strength of the individual scattered powers from both the ED and TD, which is not the case in our system. Figure 3a confirms that the TD scatters much more weakly (two orders of magnitude lower) as compared to the ED. In view of that, we would ascribe the observed scattering dip here to Fano suppression induced by the strong near field competition (mainly inside the gap region) between the AM and the CM_10_ mode.

In addition to the scattering properties shown in Fig. 3a,b, the radiation pattern can present more angular and directional information. Figure 3c,d show the far field intensity on the plane of *θ* = 90°, at *f* = 265 THz (the magnetic resonance) and *f* = 409 THz (the Fano dip), respectively. The polar plots compare contributions from the ED (red line), MD (blue line), TD (green line) and their vectorial summation (black line). Notice that in Fig. 3c the far field strength of TD is artificially amplified by 1000 times. Both radiation patterns have typical dipolar form but with different strength and orientation feature. Primarily, the scattering patterns of the ED and TD have the same angular momentum since they are both parallel to the electric component (along the *x* axis) of the incident wave and belong to the spherical dipole related to *a*_1_ 36,40,45. Regarding the scattering pattern of the MD, it is rotated by 90° with respect to that of ED and TD. The MD is parallel with the magnetic component (along the *y* axis) and belongs to the spherical dipole related to b_1_ 45. The orthogonal ED and MD are crucial for unidirectional scattering^46^,^47^,^48^ and spin-dependent photon emissions^49^. Here the designed PDA loaded with *ε*_*load*_ = 3 also exhibits the backward scattering suppression at the CM_11_ resonance (Supporting Information Fig. S3).

### Coupled oscillator model

**Figure 4:**
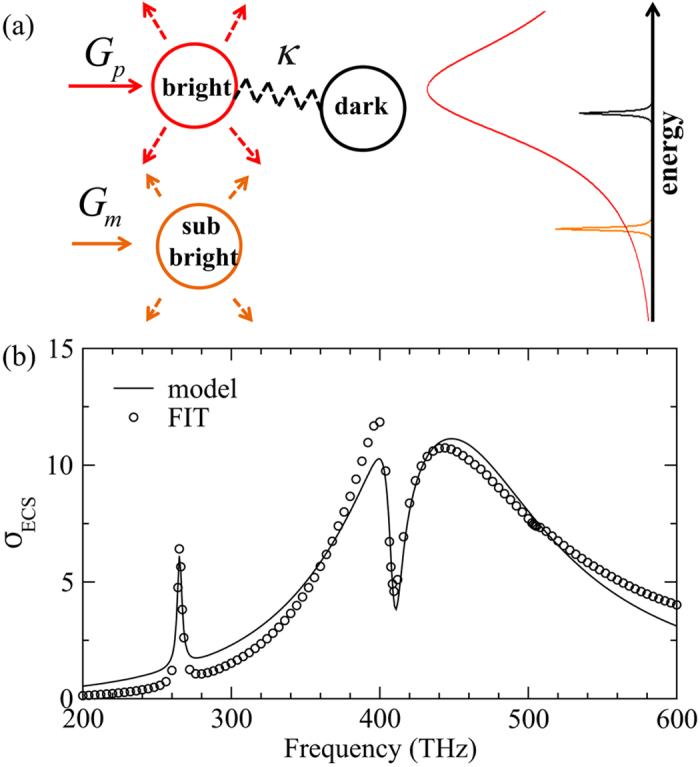
Coupled oscillator model and the fitting to numerical data. (**a**) Schematic of the coupled oscillator model for the AM-CM interactions. The coupling coefficient between the AM and CM_10_ is *κ*. Their individual resonance spectra are schematically shown at the right side. (**b**) The *σ*_*ECS*_ spectrum of the PDA numerically calculated by the FIT (circles) and the fitted result from the coupled oscillator model (line).

According to the scattering characteristics of the different modes of the PDA shown in Figs 2 and 3, we can employ a classical coupled oscillator model (COM) to understand the mode interactions in the proposed PDA^50^,^51^. Specifically, Fig. 3 shows that both the ED and MD contribute to the scattered field with different strength and channel. Therefore, it is reasonable to consider the AM as a bright mode and the CM_11_ mode as a sub-bright mode. On the other hand, the CM_10_ mode excitation is affected by the AM due to the strong near field coupling. In this regard, the CM_10_ can be considered as a dark mode despite of its weak direct excitation by the incoming wave. Figure 4a sketches the scheme of the COM where *G*_*p*_ (*G*_*m*_) represents the coupling of AM (CM_11_) to the external drive, *κ* being the coupling strength between the AM and CM_10_. The energy spectrum of each oscillator is schematically shown in the right side of Fig. 4a. The dynamic equations of this system follows^50^,^51^


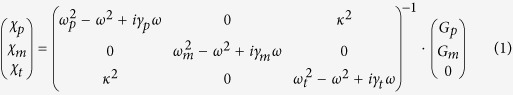


where *χ*_*p*_, *χ*_*m*_ and *χ*_*t*_ denote the responses of the bright, the sub-bright and the dark modes, respectively. The three modes have eigenfrequency *ω*_*p*_, *ω*_*m*_ and *ω*_*t*_ and intrinsic dissipation *γ*_*p*_, *γ*_*m*_ and *γ*_*t*_, respectively. In Equation (1) we have set zero coupling coefficients between *χ*_*m*_ and *χ*_*p*_ (*χ*_*t*_) since they are spectrally far away and considered to be orthogonal. The extinction spectrum of the whole coupled system is measured by −*ω*Im(*χ*_*p*_ + *χ*_*m*_) which accounts the total dissipated powers. Figure 4b shows that the model results agree qualitatively with the numerically calculated extinction spectrum *σ*_ECS_. The fitted parameters are *ω*_*p*_ = 440.38 THz, *ω*_*m*_ = 265.2 THz, *ω*_*t*_ = 410.1 THz, *γ*_*p*_ = 170.79 THz, *γ*_*m*_ = 3.828 THz, *γ*_*t*_ = 4.1 THz, *κ* = 120.78 THz, *G*_*p*_ = 1910.45 and *G*_*m*_ = 18.36. Despite of the small discrepancy between the model (line) and the FIT numerical results (circles), it is seen that the model essentially captures the features of the coexistence of scattering enhancement and suppression.

### Scattering efficiency tuning

**Figure 5:**
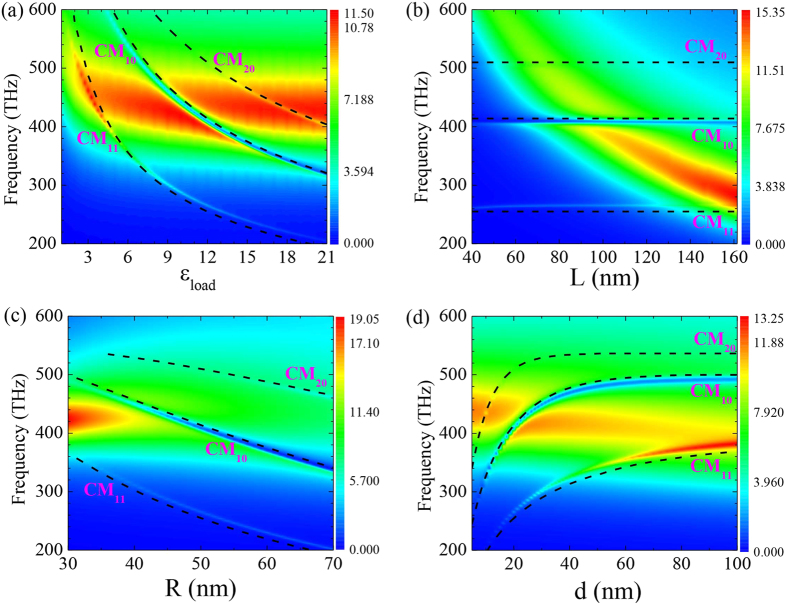
The scattering efficiency (*σ*_*SCS*_) contour in the frequency—parameter space. (a) *ε*_*load*_ from 1 to 21. (b) *L* from 40 nm to 160 nm. (c) *R* from 30 nm to 70 nm. (d) *d* from 5 nm to 100 nm. The default parameter values are the same in Fig. 2. The dashed black lines show the resonance frequency dependence on the respective design parameters, obtained by the theoretical prediction for the CM_11_, CM_10_ and CM_20_ modes.

It should be stressed that both modal field overlap (nonzero inner product of eigenmodes) and spectral overlap are necessary prerequisites to guarantee strong coupling between the AM and CM modes. In this regard, it is interesting to see how the scattering spectra are affected by these factors in realistic design. Figure 5a–d show the *σ*_*SCS*_ contour maps in the frequency band from *f* = 200 THz to 600 THz with varying *ε*_*load*_, *d*, *L* and *R*, respectively. The default parameters are kept the same as those in Fig. 2. Firstly we see that all the *σ*_*SCS*_ maps exhibit a dominating broad band coming from the AM resonance. There are also three visible narrow bands from the CM_11_, CM_10_ and CM_20_ resonances. It is clear that the CM_11_ band is a scattering enhanced one and the CM_10_ and CM_20_ bands correspond to two Fano bands with suppressed scattering. More interestingly, the strong Fano dip induced by the CM_10_ cuts through the dominating AM band in the entire frequency range. It thus introduces an anti-crossing feature, reflecting the strong interaction between the AM and the CM_10_ modes. The resonance frequencies of the CMs are theoretically predictable based on a Fabry-Perot model^41^, or approximately by applying the Neumann boundary condition for cylindrical gap SPPs at the lateral interface between the gap layer and the exterior medium for a circular MDM cavity^30^,^33^. The principle of this method is based on the fact that the resonance frequency of CM_nm_ versus 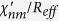 shall approximately fall on the dispersion of the gap SPPs, where 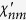 is the *n*-th zero point of the derivative of *m*-th Bessel function^30^,^33^,^52^. Therefore the resonance frequencies of the CMs are mainly determined by two factors: (1) the effective radius *R*_*eff*_ and (2) the wave vector of the gap SPP *k*_*gsp*_, both of which are closely related to the geometry and loaded material *ε*_*load*_. The design parameter dependent resonance frequencies of the CM_11_, CM_10_ and CM_20_ cavity modes are theoretically obtained and shown in Fig. 5 (black dashed lines). The theoretical prediction clearly follows the local maximum and minimum of the color contour. The small discrepancy between the theory and numerical results comes from two facts: (i) we use the transfer matrix method of a 2D multilayer, assuming ideal TM wave to get *k*_*gsp*_. But for a finite-sized structure the gap SPPs are TM-like waves; (ii) The effective radius *R*_*eff*_ ≈ *R* is an approximation and may be further modified according to the actual field distributions.

In more details, Fig. 5a shows that as the loaded material dielectric function increases from *ε*_*load*_ = 1 to 21, both the AM band and the CM bands redshift. However, the AM band is less sensitive than the CM bands with respect to the *ε*_load_ variation. In particular, for roughly *ε*_*load*_ ≥ 9, the AM band becomes flat at fixed frequency range from *f* = 400 THz to 500 THz. Thus it is possible to adjust the CM bands independently by altering the loaded material. Figure 5a further shows that for air load, e.g., with *ε*_load_ = 1, the resonance frequency of CMs is beyond *f* = 600 THz which is far from the AM band. This is one of the reasons for the absence of the AM-CM coupling effect in the literature. The red-shift of the CM bands is caused by the increased propagating constant *k*_*gsp*_ in the high dielectric constant layer. With regard to the AM, Alú *et al.* have demonstrated that the load can be applied to tune the scattering properties of the total antenna. The optical capacitance of the dielectric load is approximately *C*_load_ = *ε*_load_*πR*^2^/*d*^10^,^26^,^53^. In this view, the red-shift of the AM band is a straightforward result from the resonance condition 
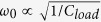
. However, this argument is valid only when we are dealing with a pure electric dipolar mode. As long as the AM is hybridized with the CMs, the situation becomes more complicated.

It is probably more feasible to tune the scattering of the antenna by adjusting its geometry parameters. Figure 5b plots the *σ*_*SCS*_ map for arm length *L* = 40 nm to 160 nm. It is seen that the AM band red-shifts rapidly while the CM bands stay nearly intact. This means that modulating the length of the metallic nanorod is an effective way to tune the AM band without affecting the CM bands much. We note that the CM bands also shift when *L* is very small because in that scenario the SPPs at the external interfaces of the metallic arms couple with the gap SPPs^30^. The CM bands are more sensitive than the AM band with respect to the radius variation, as shown in Fig. 5c. Gap cavity with bigger size favors gap SPP resonances at longer wavelength. It is also worth mentioning that the magnitude of *σ*_*SCS*_ falls quickly as *R* increases due to the reduced aspect ratio *L*/*R*. Figure 5d depicts the *σ*_*SCS*_ map as a function of the gap distance *d*. It is known that *k*_*gsp*_ − *ω* curve descends and finally converges to that of the SPP at a metal-dielectric interface when *d* is large enough. As a result, the CM bands are blue-shifted and nearly saturate when *d* ≥ 60 nm for CM_10_ and CM_20_ bands. Figure 5d further shows that the AM bands slightly red shift as *d* increases. This is inconsistent to both the nano-circuit theory^10^,^26^ and the dipole plasmon ruler equation^54^ that have blue shifting prediction for increasing *d*. We argue that these long-wavelength limit theories are valid for solid metallic particles solely supporting electric modes, which is not the case in our system. In the proposed PDAs, the AM is no longer a pure electric mode since it strongly couples to the CM_10_ mode and also couples to the CM_20_ mode. As a matter of fact, this cavity-mediation-effect was recently reported in a nancube dimer with extremely short separation^55^. The failure of the dipole approximation at shot separation is ascribed to the inhomogeneous field induced by the cavity mode, similar to those studied here. We note that when we separate the CM_10_ and AM spectrally, the AM resonance frequency blue shifts as *d* increases (Supporting Information, Figs S4 and S5).

## Discussion

In summary, we have systematically explored the anomalous scattering properties of nanoantennas made by silver nanorod homodimer that are deliberately designed to support spectrally overlapping longitudinal electric dipolar AM and transverse gap SPP CMs. It is found that the broad scattering peak of the AM is modulated by a cumulative narrow scattering peak and one or several Fano dips. By analyzing the near field characteristics, we confirm that the narrow peak is related to the CM_11_ resonance while the Fano dip corresponds to the CM_10_ resonance. By doing the multipole expansion, we have also demonstrated that CM_11_ has a prominent magnetic dipole response while the CM_10_ has a toroidal response. We believe that the physics shall be maintained and similar coupling effects should be observed even if the antenna is immersed in a different homogeneous dielectric materials or lies on a dielectric substrate. The porous anodized alumina template is probably an effective platform to fabricate the proposed structures^4^,^5^. Our findings provide more degree of freedom to manipulate the resonances in PDA, by utilizing both the AM and the CMs. The results presented here may find applications in plasmonic super-scattering and cloaking, optical sensing based on Fano resonance, spin-dependent and directional light emission, and efficient light harvest employing optical antennas.

## Methods

The full-wave electrodynamic simulations were done with a FIT solver (CST Microwave Studio 2011) and corroborated with a FEM solver (COMSOL Multiphysics 4.3a). The *σ*_*SCS*_ spectra were obtained by integrating the normal scattered Poynting over a spherical surface enclosing the PDA. In the same time, the *σ*_*ACS*_ spectra were obtained by integrating the resistive losses over the PDA volume. The extinction is defined by their sum *σ*_ECS_ = *σ*_SCS_ + *σ*_ACS_ (see Supporting Information). For all the numerical simulations, the permittivity of silver *ε*(*ω*) is taken from the experimental results of Johson and Chrisity^43^ fitted by the following Drude-Lorentz model:


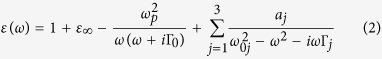


where *ω* is in unit of eV and *ε*_∞_ = 2.296, *ω*_*p*_ = 9.161 eV, Γ_0_ = 0.020 eV, *a*_1_ = 12.06, *a*_2_ = 27.67, *a*_3_ = 5.524, *ω*_01_ = 5.043 eV, *ω*_02_ = 6.171 eV, *ω*_03_ = 4.404 eV, Γ_1_ = 0.935 eV, Γ_2_ = 1.641 eV, and Γ_3_ = 0.499 eV.

## Additional Information

**How to cite this article**: Zhang, Q. *et al.* Coexistence of Scattering Enhancement and Suppression by Plasmonic Cavity Modes in Loaded Dimer Gap-Antennas. *Sci. Rep.* **5**, 17234; doi: 10.1038/srep17234 (2015).

## Supplementary Material

Supplementary Information

## References

[b1] Greffet J. J. Nanoantennas for light emission. Science 308, 1561 (2005). 15947162 10.1126/science.1113355

[b2] Novotny L. & van Hulst N. Antennas for light. Nat. Photonics 5, 83 (2011).

[b3] Xiao J. J., Huang J. P. & Yu K. W. Optical response of strongly coupled metal nanoparticles in dimer arrays. Phys. Rev. B 71, 045404 (2005).

[b4] Brintlinger T., Herzing A. A., Long J. P., Vurgaftman I., Stroud R. & Simpkins B. S. Optical dark-field and electron energy loss imaging and spectroscopy of symmetry-forbidden modes in loaded nanogap antennas. ACS Nano 9, 6222 (2015). 25961937 10.1021/acsnano.5b01591

[b5] O’Carroll D. M., Fakonas J. S., Callahan D. M., Schierhorn M. & Atwater H. A. Metal-polymer-metal split-dipole nanoantennas. Adv. Mater. 24, OP136 (2012). 22447722 10.1002/adma.201103396

[b6] Alù A. & Engheta N. Hertzian plasmonic nanodimer as an efficient optical nanoantenna. Phys. Rev. B 78, 195111 (2008).

[b7] Taminiau T. H., Stefani F. D. & van Hulst N. F. Optical nanorod antennas modeled as cavities for dipolar emitters: evolution of sub- and super-radiation modes. Nano Lett. 11, 1020 (2011). 21322590 10.1021/nl103828n

[b8] Mühlschlegel P., Eisler H. J., Martin O. J. F., Hecht B. & Pohl D. W. Resonant optical antennas. Science 308, 1607 (2005). 15947182 10.1126/science.1111886

[b9] Ward D. R., Hüser F., Pauly F., Cuevas J. C. & Natelson D. Optical rectification and field enhancement in a plasmonic nanogap. Nature Nanotech. 5, 732 (2010). 10.1038/nnano.2010.176 20852641

[b10] Alù A. & Engheta N. Tuning the scattering response of optical nanoantennas with nanocircuit loads. Nat. Photonic. 2, 307 (2008).

[b11] Pors A., Willatzen M., Albrektsen O. & Bozhevolnyi S. I. From plasmonic nanoantennas to split-ring resonators: tuning scattering strength. J. Opt. Soc. Am. B 8, 1680 (2010).

[b12] Ding W. *et al.* Understanding near/far-field engineering of optical dimer antennas through geometry modification. Opt. Express 17, 21228 (2009). 19997362 10.1364/OE.17.021228

[b13] Zhao Y., Engheta N. & Alù A. Effects of shape and loading of optical nanoantennas on their sensitivity and radiation properties. J. Opt. Soc. Am. B 28, 1266 (2011).

[b14] Nie S. & Emory S. R. Probing single molecules and single nanoparticles by surface-enhanced Raman scattering. Science 275, 1102 (1997). 9027306 10.1126/science.275.5303.1102

[b15] Curto A. G. *et al.* Unidirectional emission of a quantum dot coupled to a nanoantenna. Science 329, 930 (2010). 20724630 10.1126/science.1191922

[b16] Alù A. & Engheta N. Wireless at nanoscale: optical interconnects using matched nanoantennas. Phy. Rev. Lett. 104, 213902 (2010). 10.1103/PhysRevLett.104.213902 20867100

[b17] Large N., Abb M., Aizpurua J. & Muskens O. L. Photoconductively loaded plasmonic nanoantenna as building block for ultracompact optical switches. Nano Lett. 10, 1741 (2010). 20405903 10.1021/nl1001636

[b18] Hanke T., Krauss G., Träutlein D., Wild B., Bratschitsch R. & Leitenstorfer A. Efficient nonlinear light emission of single gold optical antennas driven by few-cycle near-infrared pulses. Phys. Rev. Lett. 103, 257404 (2009). 20366283 10.1103/PhysRevLett.103.257404

[b19] Thyagarajan K., Rivier S., Lovera A. & Martin O. J. F. Enhanced second-harmonic generation from double resonant plasmonic antennae. Opt. Express 20, 12860 (2012). 22714312 10.1364/OE.20.012860

[b20] Zhang W., Huang L., Santschi C. & Martin O. J. F. Trapping and sensing 10 nm metal nanoparticles using plasmonic dipole antennas. Nano Lett. 10, 1006 (2010). 20151698 10.1021/nl904168f

[b21] Juan M. L., Righini M. & Quidant R. Plasmon nano-optical tweezers. Nat. Photonics 5, 349 (2011).

[b22] Willingham B., Brandl D. W. & Nordlander P. Plasmon hybridization in nanorod dimers. Appl. Phys. B 93, 209 (2008).

[b23] Brown L. V., Sobhani H., Lassiter J. B., Nordlander P. & Halas N. J. Heterodimers: plasmonic properties of mismatched nanoparticle pairs. ACS Nano 4, 819 (2010). 20092361 10.1021/nn9017312

[b24] Zhang Q., Xiao J. J., Zhang X. M., Yao Y. & Liu H. Reversal of optical binding force by Fano resonance in plasmonic nanorod heterodimer. Opt. Express 21, 6601 (2013). 23482231 10.1364/OE.21.006601

[b25] Biswas S. *et al.* Plasmon-induced transparency in the visible region via self-assembled gold nanorod heterodimers. Nano Lett. 13, 6287 (2013). 24256476 10.1021/nl403911z

[b26] Alù A. & Engheta N. Input impedance, nanocircuit loading, and radiation tuning of optical nanoantennas. Phys. Rev. Lett. 101, 043901 (2008). 18764328 10.1103/PhysRevLett.101.043901

[b27] Hentschel M., Weiss T., Bagheri S. & Giessen H. Babinet to the half: coupling of solid and inverse plasmonic structures. Nano Lett. 13, 4428 (2013). 23978165 10.1021/nl402269h

[b28] Zentgraf T., Meyrath T. P., Seidel A., Kaiser S. & Giessen H. Babinet’s principle for optical frequency metamaterials and nanoantennas. Phys. Rev. B 76, 033407 (2007).

[b29] Falcone F. *et al.* Babinet principle applied to the design of metasurfaces and metamaterials. Phys. Rev. Lett. 93, 197401 (2004). 15600876 10.1103/PhysRevLett.93.197401

[b30] Zhang Q., Xiao J. J., Zhang X. M., Han D. & Gao L. Core–shell-structured dielectric–metal circular nanodisk antenna: gap plasmon assisted magnetic toroid-like cavity modes. ACS Photonics 2, 60 (2015).

[b31] Kuttge M., de Abajo F. J. G. & Polman A. Ultrasmall mode volume plasmonic nanodisk resonators. Nano Lett. 10, 1537 (2010). 19813755 10.1021/nl902546r

[b32] Kwon S. H. Deep subwavelength plasmonic whispering gallery-mode cavity. Opt. Express 20, 24918 (2012). 23187259 10.1364/OE.20.024918

[b33] Minkowski F., Wang F., Chakrabarty A. & Wei Q. H. Resonant cavity modes of circular plasmonic patch nanoantennas. App. Phys. Lett. 104, 021111 (2014).

[b34] Dong Z. G. *et al.* All-optical Hall effect by the dynamic toroidal moment in a cavity-based metamaterial. Phys. Rev. B 87, 245429 (2013).

[b35] Zhang Q., Xiao J. J. & Wang S. L. Optical characteristics associated with magnetic resonance in toroidal metamaterials of vertically coupled plasmonic nanodisks. J. Opt. Soc. Am. B 31, 1103 (2014).

[b36] Miroshnichenko A. E. *et al.* Nonradiating anapole modes in dielectric nanoparticles. Nat. Commun. 6, 8069 (2015). 26311109 10.1038/ncomms9069 PMC4560796

[b37] Liu W., Zhang J., Lei B. & Hu H. Toroidal dipole induced transparency of core-shell nanoparticles. Laser Photon. Rev., 9, 564 (2015).

[b38] Liu W., Zhang J., Lei B., Hu H. & Miroshnichenko A. E. Invisible nanowires with interfering electric and toroidal dipoles. Opt. Lett. 40, 2293 (2015). 26393722 10.1364/OL.40.002293

[b39] Fedotov V. A., Rogacheva A. V., Savinov V., Tsai D. P. & Zheludev N. I. Resonant transparency and non-trivial non-radiating excitations in toroidal metamaterials. Sci. Rep. 3, 2967 (2013). 24132231 10.1038/srep02967 PMC3797985

[b40] Zhang X. L., Wang S. B., Lin Z., Sun H. B. & Chan C. T. Optical force on toroidal nanostructures: toroidal dipole versus renormalized electric dipole. Phys. Rev. A 92, 043804 (2015).

[b41] Esteban R. *et al.* The morphology of narrow gaps modifies the plasmonic response. ACS Photonics 2, 295 (2015).

[b42] CST Microwave Studio 2011 (http://www.cst.com).

[b43] Johson P. B. & Christy R. W. The optical constants of noble metals. Phys. Rev. B 6, 4370 (1972).

[b44] COMSOL Multiphysics 4.3a (http://www.comsol.com).

[b45] Grahn P., Shevchenko A. & Kaivola M. Electromagnetic multipole theory for optical nanomaterials. New J. Phys. 14, 093033 (2012).

[b46] Staude I. *et al.* Tailoring directional scattering through magnetic and electric resonances in subwavelength silicon nanodisks. ACS Nano 7, 7824 (2013). 23952969 10.1021/nn402736f

[b47] Liu W., Miroshnichenko A. E., Neshev D. N. & Kivshar Y. S. Broadband unidirectional scattering by magneto-electric core–shell nanoparticles. ACS Nano 6, 5489 (2012). 22545872 10.1021/nn301398a

[b48] Decker M. *et al.* High-efficiency dielectric Huygens’ surfaces. Adv. Optical Mater. 3, 813 (2015).

[b49] Kruk S. S. *et al.* Spin-polarized photon emission by resonant multipolar nanoantennas. ACS Photonics 1, 1218 (2015).

[b50] Zhang K. *et al.* Dual-mode electromagnetically induced transparency and slow light in a terahertz metamaterial. Opt. Lett. 39, 3539 (2014). 24978531 10.1364/OL.39.003539

[b51] Lovera A., Gallinet B., Nordlander P. & Martin O. J. F. Mechanisms of Fano resonances in coupled plasmonic systems. ACS Nano 7, 4527 (2013). 23614396 10.1021/nn401175j

[b52] Zhang X. M., Xiao J. J., Zhang Q., Li L. M. & Yao Y. Plasmonic TM-like cavity modes and the hybridization in multilayer metal-dielectric nanoantenna. Opt. Express 23, 16122 (2015). 26193585 10.1364/OE.23.016122

[b53] Engheta N., Salandrino A. & Alù A. Circuit elements at optical frequencies: nanoinductors, nanocapacitors, and nanoresistors. Phys. Rev. Lett. 95, 095504 (2005). 16197226 10.1103/PhysRevLett.95.095504

[b54] Jain P. K., Huang W. & Ei-Sayed M. A. On the universal scaling behavior of the distance decay of plasmon coupling in metal nanoparticle pairs: a plasmon ruler equation. Nano Lett. 7, 2080 (2007). 10.1021/nl071496m 17676810

[b55] Bordley J. A., Hooshmand N. & Ei-Sayed M. A. The coupling between gold or silver nanocubes in their homo-dimers: a new coupling mechanism at short separation distances. Nano Lett. 15, 3391 (2015). 25844929 10.1021/acs.nanolett.5b00734

