# Supplementary material for: Coexistence of Scattering Enhancement and Suppression by Plasmonic Cavity Modes in Loaded Dimer Gap-Antennas
Source: Sci Rep. 2015 Nov 27;5:17234. doi: 10.1038/srep17234 (PMC4661512; doi:10.1038/srep17234)
Supplement: Supplementary Information [file srep17234-s1.pdf]

Supporting Information of

"Coexistence of Scattering Enhancement and Suppression by Plasmonic Cavity Modes in Loaded Dimer Gap-Antennas"

Qiang Zhang,[1] Jun-Jun Xiao,[1,*] Meili Li,[1] Dezhuan Han,[2] and Lei Gao[3,4]

[1]*College of Electronic and Information Engineering, Shenzhen Graduate School, Harbin Institute of Technology, Xili, Shenzhen 518055, China*

[2]*Department of Applied Physics, Chongqing University, Chongqing 400044, China*

[3]*College of Physics, Optoelectronics and Energy of Soochow University, & Collaborative Innovation Center of Suzhou Nano Science and Technology, Soochow University, Suzhou 215006, China*

[4]*Jiangsu Key Laboratory of Thin Films, Soochow University, Suzhou 215006, China.*

[*]*Corresponding author:* eiexiao@hitsz.edu.cn

I. Multipole decomposition.

The multipole decompositions are accomplished in both the Cartesian basis (source-representation) and the spherical basis (field-representation). The irreducible Cartesian mutipole moments (using the notation of $e^{-i\omega t}$ for electromagnetic waves) are evaluated by [1-5]

$$
\begin{aligned}
P_\alpha &= -\frac{1}{i\omega}\int J_\alpha d^3r \\
M_\alpha &= \frac{1}{2c}\int [\mathbf{r}\times\mathbf{J}]_\alpha \, d^3r \\
T_\alpha &= \frac{1}{10c}\int \left[(\mathbf{r}\cdot\mathbf{J})r_\alpha - 2r^2 J_\alpha\right] d^3r \\
Q^e_{\alpha\beta} &= -\frac{1}{i\omega}\int \left[r_\alpha J_\beta + J_\alpha r_\beta - \frac{2}{3}\delta_{\alpha\beta}(\mathbf{r}\cdot\mathbf{J}) \right] d^3r \\
Q^m_{\alpha\beta} &= \frac{1}{3c}\int \left[[\mathbf{r}\times\mathbf{J}]_\alpha r_\beta + r_\alpha [\mathbf{r}\times\mathbf{J}]_\beta \right] d^3r
\end{aligned} \tag{1}
$$

where $\mathbf{P}$ is the electric dipole moment, $\mathbf{M}$ the magnetic dipole moment, $\mathbf{T}$ the toroidal dipole moment, $\mathbf{Q}^e$ the component of electric quadrupole moment, and $\mathbf{Q}^m$ is the magnetic quadrupole moment, $\mathbf{J}$ is the induced current density, $\mathbf{r}$ is distance vector from the origin (gap center) to point (x, y, z) in the Cartesian coordinate system and the subscripts $\alpha, \beta = x, y, z$ represent the corresponding components. In structures with canonical geometry, for example sphere or cylindrical particles, it is possible to get the explicit resonant conditions from Equation (1) since the current distribution can be analytically presented by the Mie theory. However, that is basically impossible in non-canonical structures.

The time-averaged scattered powers of the mutipoles can be written as the following summation:

$$
I = \frac{1}{8\pi\varepsilon_0}\left(\frac{2\omega^4}{3c^3}|\mathbf{P}|^2 + \frac{2\omega^4}{3c^3}|\mathbf{M}|^2 + \frac{4\omega^5}{3c^4}\mathrm{Im}(\mathbf{P}\cdot\mathbf{T}^*) + \frac{2\omega^6}{3c^5}|\mathbf{T}|^2 + \frac{\omega^6}{20c^5}\sum|\mathbf{Q}^e|^2 + \frac{\omega^6}{20c^5}\sum|\mathbf{Q}^m|^2\right) \tag{2}
$$

The third term in Equation (4) is the scattered power from the ED-TD cross term, where the '*' denotes the complex conjugate. The spherical electric dipole moment $\mathbf{P}_{\mathrm{sph}} = \varepsilon_0 E_0 6\pi i a_1 / k^3$, the Cartesian electric dipole moment $\mathbf{P}$ and toroidal dipole moment $\mathbf{T}$ are actually related by $\mathbf{P}_{sph} = \mathbf{P} + ik\mathbf{T} + \ldots$, where a_1 is the spherical dipole scattering coefficient， ε_0 the permittivity of the vacuum, E_0 the amplitude of the incident wave, k the wave vector and '…' represents

higher-order correcting terms. The ED-TD cross term is part of the scattered power from $\mathbf{P}_{\text{sph}}$ that is proportional to $\left|\mathbf{P}_{sph}\right|^2 = \left|\mathbf{P} + ik\mathbf{T}\right|^2$ when the higher-order correcting terms are negligible [3, 6]. And the scattering efficiency in vacuum is defined as

$$\sigma_{\text{scs}} = \frac{C_{\text{scs}}}{2R(2L+d)} \tag{3}$$

where the scattering cross section reads

$$C_{SCS} = \frac{2Z_0 \mathrm{I}}{\left|E_0\right|^2} \tag{4}$$

In Equation (6), Z_0 is the wave impedance of vacuum and E_0 is the amplitude of incident plane wave.

The scattered electric far field from $\mathbf{P}$, $\mathbf{M}$ and $\mathbf{T}$ follows [1,7]

$$\mathbf{E}_{far} = \frac{k^2}{4\pi\varepsilon_0}\frac{e^{ikr}}{r}\left[\hat{\mathbf{n}}\times(\mathbf{P}\times\hat{\mathbf{n}}) + (\mathbf{M}\times\hat{\mathbf{n}}) + ik\cdot\hat{\mathbf{n}}\times(\mathbf{T}\times\hat{\mathbf{n}})\right] \tag{5}$$

where k is the wave vector in the background medium, $\hat{\mathbf{n}}$ is the unit vector denoting the radiation direction. In Fig. 3c and 3d, the far field intensity was calculated at the surface of a sphere of $r = 1$ m, origined in the antenna center.

The spherical multipole moments are obtained by calculating the scattering coefficients through the following field projection [8,9]

$$\begin{aligned} a_{lm} &= \frac{(-i)^{l+1} kr}{h_l^{(1)}(kr) E_0 \left[\pi(2l+1)l(l+1)\right]^{\frac{1}{2}}} \int_0^{2\pi}\int_0^{\pi} Y_{lm}^*(\theta,\phi)\hat{\mathbf{r}}\cdot\mathbf{E}_s(\mathbf{r})\sin(\theta)\, d\theta d\phi \\ b_{lm} &= \frac{(-i)^{l} \eta kr}{h_l^{(1)}(kr) E_0 \left[\pi(2l+1)l(l+1)\right]^{\frac{1}{2}}} \int_0^{2\pi}\int_0^{\pi} Y_{lm}^*(\theta,\phi)\hat{\mathbf{r}}\cdot\mathbf{H}_s(\mathbf{r})\sin(\theta)\, d\theta d\phi \end{aligned} \tag{6}$$

where Y_{lm} and $h_l^{(1)}$ are the scalar spherical harmonics and the spherical Hankel function of the first kind, respectively; $\mathbf{E}_s$ ($\mathbf{H}_s$) is the scattered electric field (magnetic field), E_0 is the amplitude of the incident wave, η is the impedance of the surrounding media, and $\hat{\mathbf{r}}$ is the unit directional vector. Then the scattering cross sections by the spherical dipoles a_1 and b_1 read:

$$C_{SCS} = \frac{3\pi}{k^2} \sum_{m=-1}^{1} \left[\left| a_{1m} \right|^2 + \left| b_{1m} \right|^2 \right] \tag{7}$$

II. Theoretical resonance frequency of the cavity modes.

In cylindrical coordinate, the field of the gap SPPs at the dielectric layer has the ansatz form [10-12]:

$$E_z\left(\rho, \phi, z\right) = \mathrm{a(z)} J_m(k_{gsp}\rho)\, \mathrm{e}^{im\phi} \tag{8}$$

where k_{gsp} is the wave vector of the gap SPPs in the MIM structure, a(z) the mode profile in z direction, and m is the azimuthal number. At the dielectric layer, the electric field of the gap SPPs at the lateral edge reaches the locale maximum, i.e., the Neumann boundary condition $\partial E_z(R_{eff})/\partial\rho = 0$ shall be satisfied. Combing with equation (10), the resonance condition then reads

$$k_{gsp}(\omega) = \frac{\chi'_{nm}}{R_{eff}} \tag{9}$$

where χ'_{nm} denoting the n-th root of the first-order derivation of the m-th Bessel function, R_{eff} is the appropriate effective radius depending on how strong the field leaks out the circumferences. After obtaining the dispersion relation of the gap SPPs $k_{gsp}(\omega)$, the resonance frequency can be extracted from equation (11). In this study, $k_{gsp}(\omega)$ is obtained from the divergence of the reflection coefficient calculated by the transfer matrix method of a 2D metal-dielectric-metal three layer system [12]. The black dashed lines in Fig. 5 obtained in this way match the numerical CM bands well by applying $R_{eff} \approx R$, meaning extremely weak leakage of the cavity mode fields.

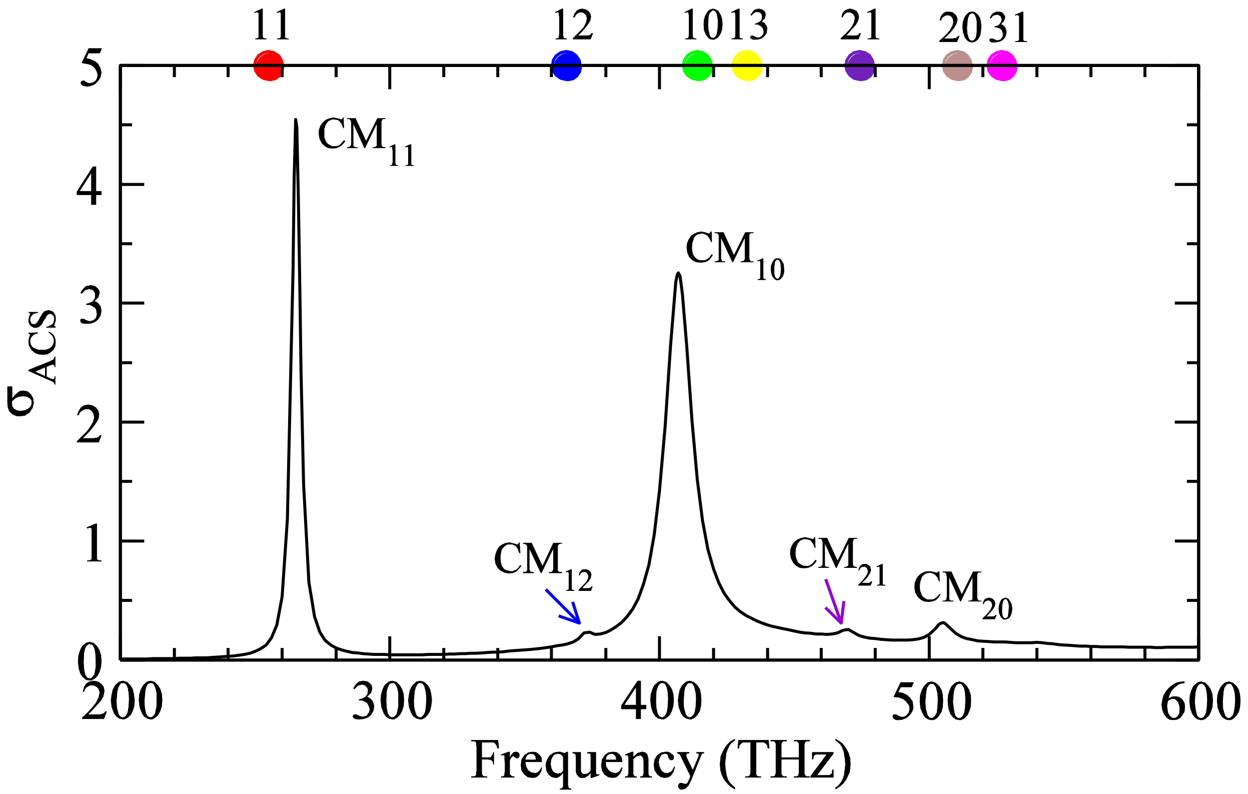

Figure S1. The absorption spectrum σ_{ACS} of the PDA studied in Figure 2. The color dots on the top x-axis mark the theoretically predicted resonance position of the seven CMs labeled with radial and azimuthal numbers 'nm'. The blue arrow and the indigo arrow indicate the peaks of CM_{12} and CM_{21} resonances, respectively. The remaining CM_{13} and CM_{31} resonance are too weak to have visible features even in the absorption spectrum. Note that all these CMs can be excited efficiently in a circular patch MIM resonator, partially due to the absence of the AM in the same frequency range [10].

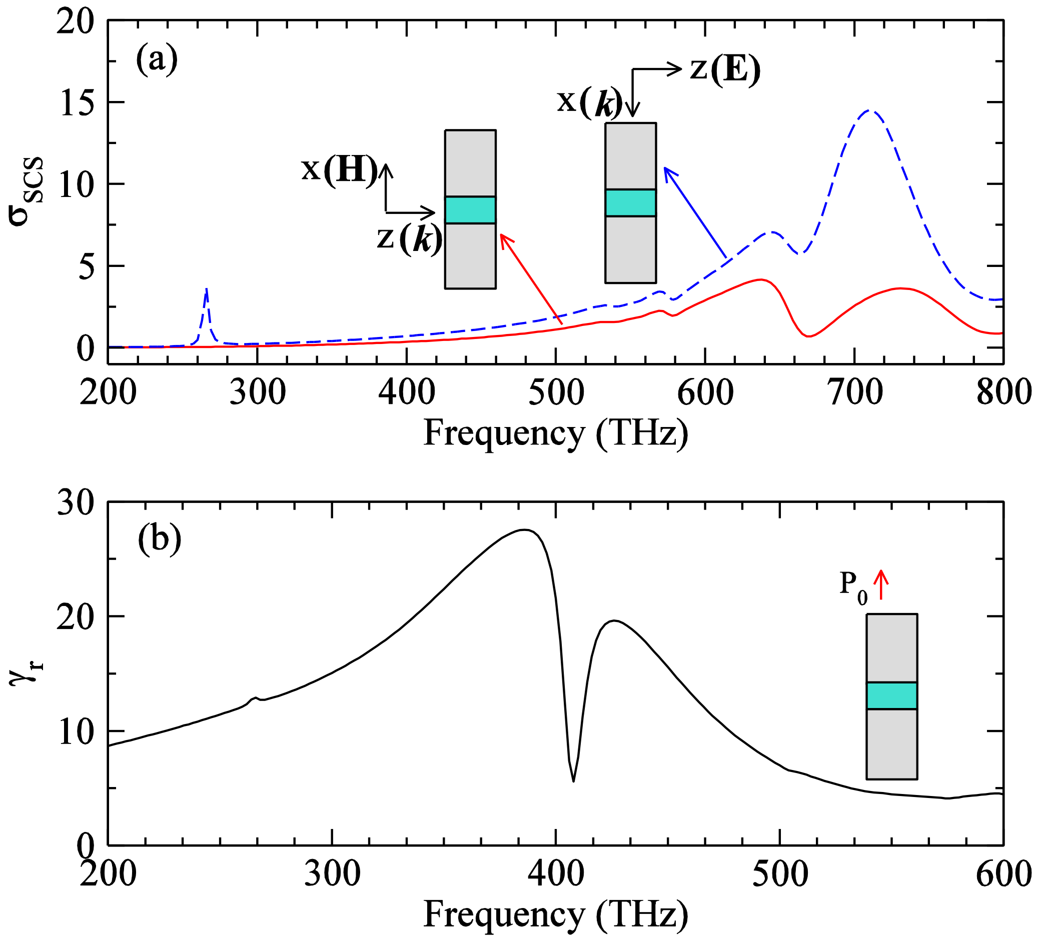

Figure S2. (a) The scattering spectrum σ_{SCS} of the PDA excited by an incoming plane wave in the other two configurations (see insets): (i) $\boldsymbol{k}$ along the z-axis, **H** along the x-axis (red solid line) and (ii) $\boldsymbol{k}$ along the x-axis, **E** along the z-axis (blue dashed line). The longitudinal electric AM is not excitable in these two configurations and the most prominent scattering peak at the high frequency is actually associated with the transverse electric dipole mode [13]. (b) The radiation decay rate γ_r [14,15] of an active electric dipole placed near the end of the PDA (the red arrow of the insert). Obviously, the radiation of the dipole is strongly modulated near the cumulative peak and the suppressed dip.

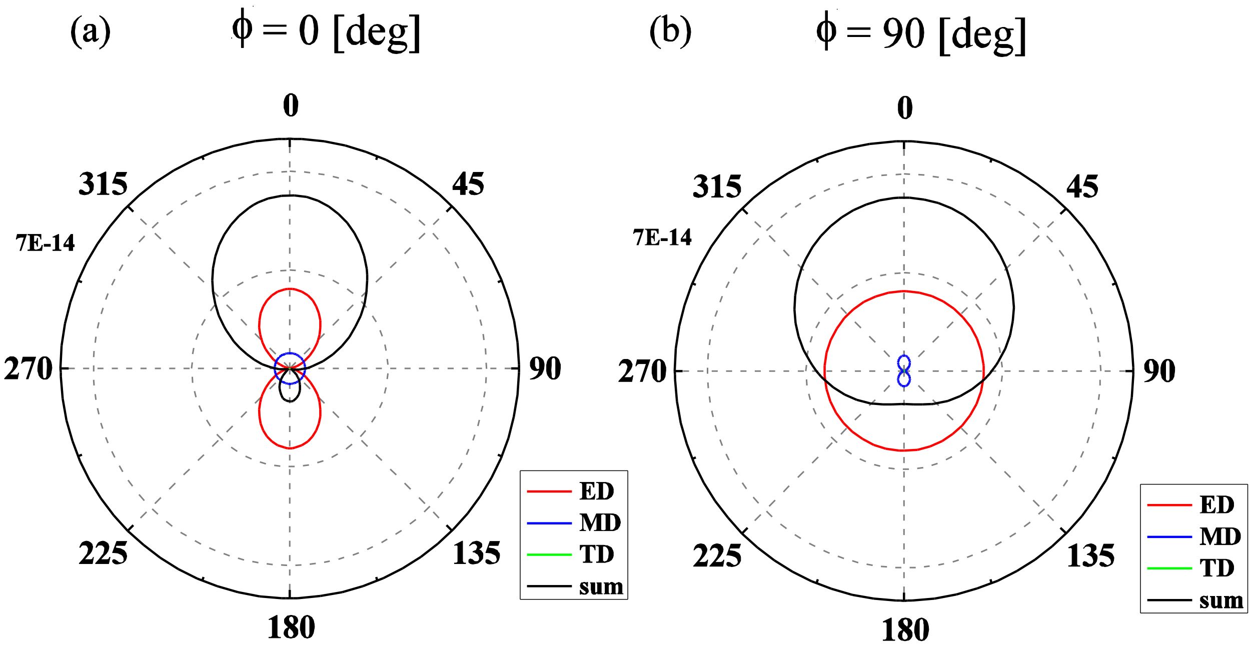

Figure S3. The radiation pattern $\left|\mathbf{E}_{far}(\theta,\phi)\right|^2$ at the plane of (a) $\phi = 0^\circ$ and (b) $\phi = 90^\circ$ for a PDA with $L = 90\,\text{nm}$, $R = 50$ nm, $d = 20$ nm, and $\varepsilon_{load} = 3$. The resonant frequency is $f = 462$ THz (i.e., the scattering peak shown in Figure 1c). At this frequency, the CM_{11} and AM both resonantly contribute to the scattered far field (red and blue lines), but with different magnitudes. Their vectorial summation (black lines) shows that the backward scattering is substantially suppressed, although not completely vanishing.

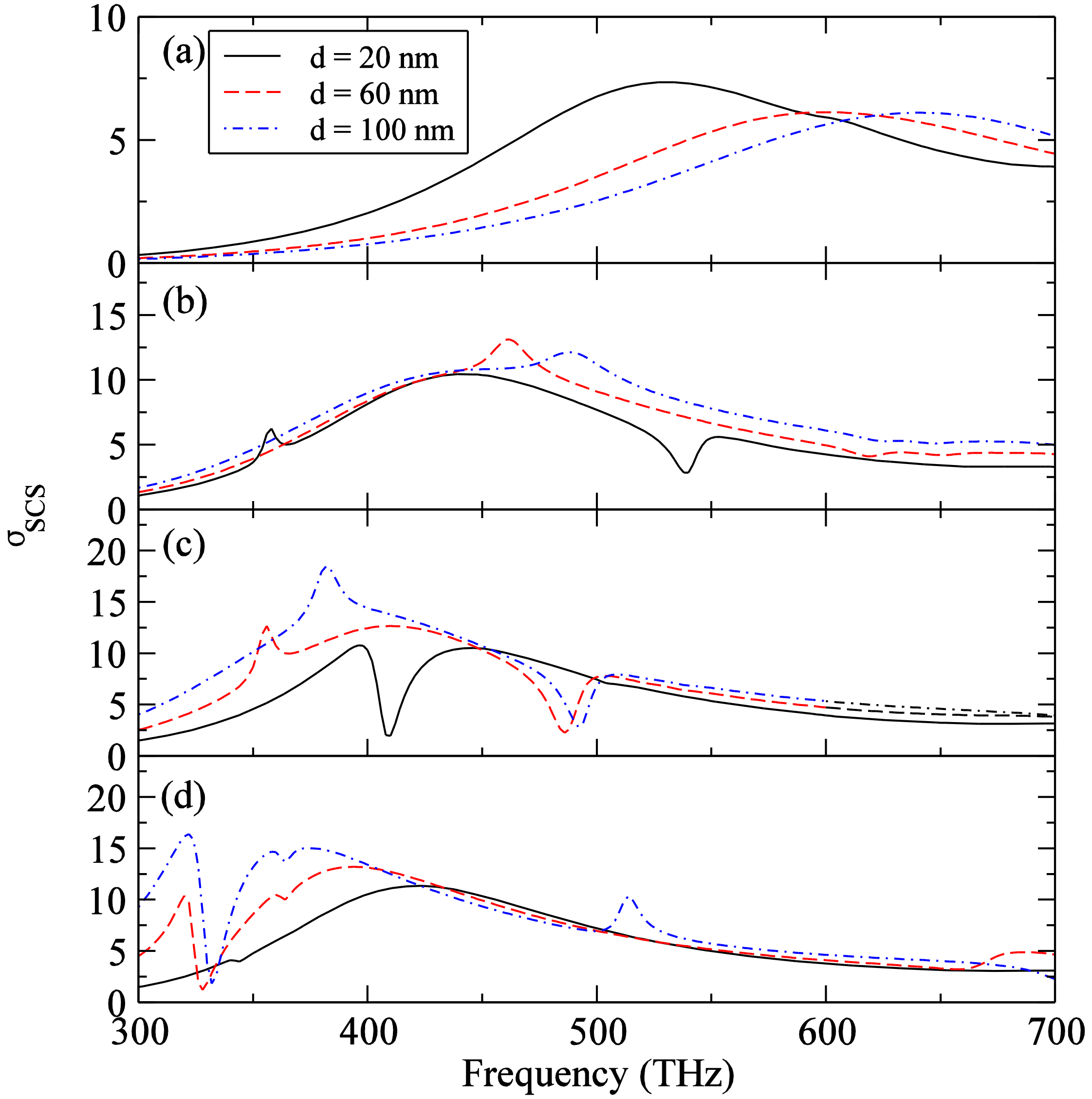

Figure S4. Evolutions of the σ_{scs} spectrum for different gap distance d of the PDAs with (a) $\varepsilon_{load}=1$, (b) $\varepsilon_{load}=6$, (c) $\varepsilon_{load}=12$, and (d) $\varepsilon_{load}=30$. For $\varepsilon_{load}=1$, the AM is not affected by the CMs and the AM resonance frequency blue shifts for increasing d as predicted by the plasmon ruler equation and the hybridization model [16,17]. When ε_{load} increases, the CMs are tuned to overlap with the broad AM spectrally and couple with the AM, deteriorating the blue-shift trend. Especially, when the AM is strongly modified by the Fano dip in (c) and (d), its peak is contrarily red-shifted as the separation distance d increases.

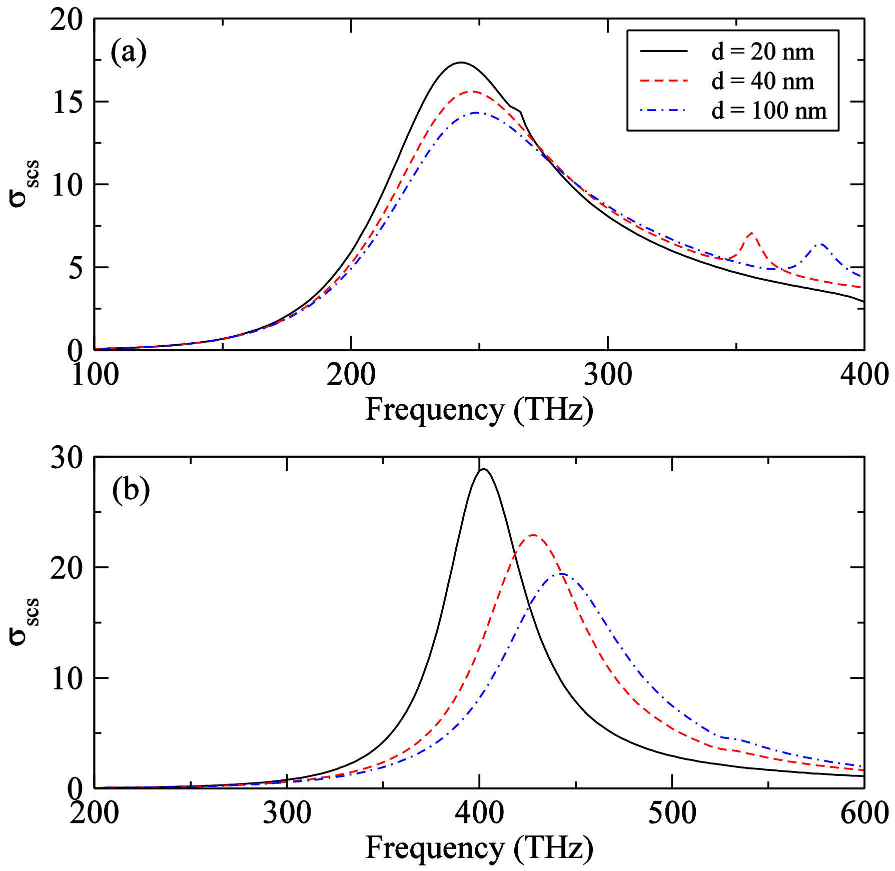

Figure S5. Evolution of the σ_{scs} spectrum for different gap distance d when (a) the AM is moved far away from CM_{10} by increasing L to 200 nm, and (b) CM_{10} is moved far away from the AM by decreasing R to 20 nm. Again, the AM becomes blue shifted as d increases as long as the AM and CM_{10} are separated spectrally.

References:

[1] Liu, W.; Zhang, J.; Lei, B.; Hu, H. Toroidal Dipole Induced Transparency of Core-Shell Nanoparticles. *Laser Photon. Rev.*, doi:10.1002/lpor.201500102, **2015**.

[2] Liu, W.; Zhang, J.; Lei, B.; Hu, H.; Miroshnichenko, A. E. Invisible Nanowires with Interfering Electric and Toroidal Dipoles. *Opt. Lett.* **2015**, *40*, 2293-2296.

[3] Fedotov, V. A.; Rogacheva, A. V.; Savinov, V.; Tsai, D. P.; Zheludev, N. I. Resonant Transparency and Non-Trivial Non-Radiating Excitations in Toroidal Metamaterials. *Sci. Rep.* **2013**, *3*, 2967.

[4] Zhang, X. L.; Wang, S. B.; Lin, Z.; Sun, H. B.; Chan, C. T. Optical Force on Toroidal Nanostructures: Toroidal Dipole versus Renormalized Electric Dipole. arXiv:1506. 01574 [physics. optics], **2015**.

[5] Radescu, E. E.; Vaman, G. Exact Calculation of the Angular Momentum Loss, Recoil Force, and Radiation Intensity for an Arbitrary Source in terms of Electric, Magnetic, and Toroid Multipoles. *Phys. Rev. E* **2002**, *65*, 046609.

[6] Liu, W.; Shi, J.; Lei, B.; Hu, H.; Miroshnichenko, A. E.; Efficient Excitation and Tuning of Toroidal Dipoles within Individual Homogenous Nanoparticles. *Opt. Express* **2015**, 23, 24738-24747.

[7] Chen, J.; Ng, J.; Lin, Z.; Chan, C. T. Optical Pulling Force. *Nat. Photonics* **2011**, *5*, 531-534.

[8] Grahn, P.; Shevchenko, A.; Kaivola, M. Electromagnetic Multipole Theory for Optical Nanomaterials. *N. J. Phys.* **2012**, *14*, 093033.

[9] Bohren, C. F.; Huffman, D. R. *Absorption and Scattering of Light by Small Particles.* John Wiley & Sons Inc: New York, **1983**.

[10] Zhang, Q.; Xiao, J. J.; Zhang, X. M.; Han, D.; Gao, L. Core–Shell-Structured Dielectric–Metal Circular Nanodisk Antenna: Gap Plasmon Assisted Magnetic Toroid-like Cavity Modes. *ACS Photonics* **2015**, *2*, 60-65.

[11] Minkowski, F.; Wang, F.; Chakrabarty, A.; Wei, Q. H. Resonant Cavity Modes of Circular Plasmonic Patch Nanoantennas. *App. Phys. Lett.* **2014**, *104*, 021111.

[12] Zhang, X. M.; Xiao, J. J.; Zhang, Q.; Li, L. M.; Yao, Y. Plasmonic TM-like Cavity Modes and the Hybridization in Multilayer Metal-Dielectric Nanoantenna. *Opt. Express* **2015**, *23*, 16122-16132.

[13] Brintlinger, T.; Herzing, A. A.; Long, J. P.; Vurgaftman, I.; Stroud, R.; Simpkins, B. S. Optical Dark-Field and Electron Energy Loss Imaging and Spectroscopy of Symmetry-Forbidden Modes in Loaded Nanogap Antennas. *ACS Nano* **2015**, *9*, 6222-6232.

[14] Zhang, X. M.; Xiao, J. J.; Zhang, Q. Interaction Between Single Nano-emitter and Plasmonic Disk-ring Nanostructure with Multiple Fano Resonaces. *J. Opt. Soc. Am. B* **2014**, *31,* 2193-2200.

[15] Zhang, X. M.; Xiao, J. J.; Zhang, Q.; Li, L. M.; Yao, Y. Plasmonic TM-like Cavity Modes and the Hybridization in Multilayer Metal-dielectric Nanoantenna. *Opt. Express*

2015, *23*, 16122-16132.

[16] Jain, P. K.; Huang, W.; Ei-Sayed, M. A. On the Universal Scaling Behavior of the Distance Decay of Plasmon Coupling in Metal Nanoparticle Pairs: A Plasmon Ruler Equation. *Nano Lett.* **2007**, *7*, 2080-2088.

[17] Willingham, B.; Brandl, D. W.; Nordlander, P. Plasmon Hybridization in Nanorod Dimers. *Appl. Phys. B* **2008**, *93*, 209-216.
